# Rhinovirus infection induces secretion of endothelin-1 from airway epithelial cells in both in vitro and in vivo models

**DOI:** 10.1186/s12931-023-02510-6

**Published:** 2023-08-19

**Authors:** Alane Blythe C. Dy, Jason Girkin, Antonella Marrocco, Adam Collison, Chimwemwe Mwase, Michael J. O’Sullivan, Thien-Khoi N. Phung, Joerg Mattes, Cynthia Koziol-White, James E. Gern, Yury A. Bochkov, Nathan W. Bartlett, Jin-Ah Park

**Affiliations:** 1 Program in Molecular and Integrative Physiological Sciences, Department of Environmental Health, Harvard T.H. Chan School of Public Health, 665 Huntington Ave, Boston, MA SPH1-315 USA; 2 College of Health, Medicine and Wellbeing, University of Newcastle and Hunter Medical Research Institute, New Lambton Heights, Australia; 3 Robert Wood Johnson Medical School, Rutgers University, New Brunswick, NJ USA; 4 Department of Pediatrics, School of Medicine and Public Health, University of Wisconsin-Madison, Madison, WI USA

**Keywords:** Rhinovirus infection, Epithelial endothelin-1, Asthma exacerbations, Bronchoconstriction, Mechanical compression

## Abstract

**Background:**

Rhinovirus (RV) infection of airway epithelial cells triggers asthma exacerbations, during which airway smooth muscle (ASM) excessively contracts. Due to ASM contraction, airway epithelial cells become mechanically compressed. We previously reported that compressed human bronchial epithelial (HBE) cells are a source of endothelin-1 (ET-1) that causes ASM contraction. Here, we hypothesized that epithelial sensing of RV by TLR3 and epithelial compression induce ET-1 secretion through a TGF-β receptor (TGFβR)-dependent mechanism.

**Methods:**

To test this, we used primary HBE cells well-differentiated in air–liquid interface culture and two mouse models (ovalbumin and house dust mite) of allergic airway disease (AAD). HBE cells were infected with RV-A16, treated with a TLR3 agonist (poly(I:C)), or exposed to compression. Thereafter, *EDN1 (*ET-1 protein-encoding gene) mRNA expression and secreted ET-1 protein were measured. We examined the role of TGFβR in ET-1 secretion using either a pharmacologic inhibitor of TGFβR or recombinant TGF-β1 protein. In the AAD mouse models, allergen-sensitized and allergen-challenged mice were subsequently infected with RV. We then measured ET-1 in bronchoalveolar lavage fluid (BALF) and airway hyperresponsiveness (AHR) following methacholine challenge.

**Results:**

Our data reveal that RV infection induced *EDN1* expression and ET-1 secretion in HBE cells, potentially mediated by TLR3. TGFβR activation was partially required for ET-1 secretion, which was induced by RV, poly(I:C), or compression. TGFβR activation alone was sufficient to increase ET-1 secretion. In AAD mouse models, RV induced ET-1 secretion in BALF, which positively correlated with AHR.

**Conclusions:**

Our data provide evidence that RV infection increased epithelial-cell ET-1 secretion through a TGFβR-dependent mechanism, which contributes to bronchoconstriction during RV-induced asthma exacerbations.

## Introduction

Asthma is a chronic airway disease characterized by airway inflammation, progressive airway remodeling, and acute exacerbations [1, 2]. In patients with asthma, respiratory viral infections are common triggers of exacerbations, most frequently attributed to rhinovirus (RV) [3–7]. During an acute exacerbation, excessive contraction of airway smooth muscle (ASM) that narrows airways (bronchoconstriction) is a major pathological event contributing to the severity of asthma symptoms [1, 8, 9]. However, mechanisms underlying the link between RV infection of airway epithelial cells and bronchoconstriction remain poorly understood. It is unknown whether bronchoconstriction is directly caused by mediators secreted from infected airway epithelial cells, even prior to the recruitment and activation of inflammatory immune cells. To address this knowledge gap, we focused on endothelin-1 (ET-1) that is secreted from human bronchial epithelial (HBE) cells because ET-1 is a potent bronchoconstrictor that causes airway narrowing [10].

Since the identification of ET-1 in endothelial cells, its regulatory mechanisms and biological functions have been extensively studied in vascular endothelial cells in the context of pulmonary arterial hypertension (PAH) [11, 12]. However, the regulatory mechanisms of ET-1 expression may depend on the context of the disease and the specific cell types [12, 13]. In the lung, besides PAH, ET-1 is most well-recognized in the context of pulmonary fibrosis, where TGF-β plays a prominent role in the pathogenesis of the disease [12–14]. In patients with idiopathic pulmonary fibrosis (IPF), ET-1 concentration is increased in serum and bronchoalveolar lavage fluid (BALF), and expression of ET-1 is increased in bronchial epithelial cells and type II alveolar epithelial cells [15]. In an in vitro study using rat type II alveolar epithelial cells, ET-1 secretion is induced by TGF-β 1 [16], which could be a link between ET-1 and IPF. Like in IPF, in patients with asthma, both TGF-β1 and ET-1 are increased in serum and BALF [14, 17–19]. The expression of TGF-β1 and ET-1 in bronchial epithelial cells is positively correlated with disease severity, airway remodeling, and airflow obstruction [20–22]. However, mechanisms underlying increased ET-1 expression, as well as the role of TGF-β1 in ET-1 expression in HBE cells, remain unknown. Using an in vitro system that mimics the effect of mechanical compression on airway epithelial cells during bronchoconstriction, we previously demonstrated that mechanical compression significantly induces ET-1 secretion from HBE cells [23, 24]. Our data also revealed that this epithelial cell-derived ET-1 induces ASM contraction [24]. To extend our previous findings and identify molecular mechanisms behind increased ET-1 expression in bronchial epithelial cells, we hypothesized that RV infection induces ET-1 expression and results in ET-1 secretion. To examine mechanisms of RV-induced ET-1 secretion from bronchial epithelial cells, we used primary HBE cells differentiated in air–liquid interface (ALI) culture. Then, to determine RV-induced ET-1 in BALF and its correlation with airway hyperresponsiveness (AHR), we used mouse models (ovalbumin and house dust mite) of allergic airway disease (AAD).

## Materials and methods

### Culture of primary human bronchial epithelial cells

Primary HBE cells at passage 1 were obtained from the Cystic Fibrosis Center Tissue Procurement and Cell Culture Core, under the protocol (No. 03-1396) approved by the Biomedical Institutional Review Board at the University of North Carolina, Chapel Hill. As previously published [24–27], primary HBE cells at passage 2 were cultured from donors with no history of lung disease and differentiated in ALI culture. HBE cells were cultured and maintained using a 1:1 mixture of Dulbecco’s modified Eagle’s medium (DMEM, Corning Inc., Corning, NY) and bronchial epithelial cell growth basal medium (BEBM, Lonza, Basel, Switzerland), supplemented with bronchial epithelial growth medium (BEGM) SingleQuot kit (Lonza, Basel, Switzerland), nystatin (20 units/ml, Sigma Aldrich, St. Louis, MO), all-trans-retinoic acid (50 nM, Sigma Aldrich, St. Louis, MO), and bovine serum albumin (1.5 µg/ml, Sigma Aldrich, St. Louis, MO). Upon reaching confluence after 5–7 days in submerged culture, media from the apical side was removed to establish the air–liquid interface (ALI) and cells cultured for an additional 14–16 days, to achieve well-differentiated phenotypes as presented previously [26]. Prior to infection with human rhinovirus A16 (RV-A16) or exposure to stimuli, cells were maintained for 20 h in minimal medium, which is depleted of hydrocortisone, epidermal growth factor, and bovine pituitary extract. For each experimental condition, differentiated HBE cells were used in duplicate (ie. two transwells per control or any treatment condition for the cells from each donor).

### Infection of HBE cells by RV-A16

RV-A16 was grown by infection of H1-HeLa cells (CRL-1958) and purified by ultracentrifugation (200,000 × *g*, for two hours, at 10 °C) through 30% (w/v) sucrose cushion as described [28, 29]. Virus pellet was resuspended in PBS containing 0.01% BSA. Well-differentiated HBE cells were apically infected with RV-A16 at 1 $\times$ 10^5^ ~ 10^7^ plaque-forming units (PFU) per transwell with 1.1 cm^2^ surface area (10^6^ PFU $\approx$ multiplicity of infection (MOI) of 1) for four hours, as previously described [30, 31]. Uninfected cells were treated the same as infected cells, except for the presence of the virus in apical media.

### In vitro exposure of HBE cells to poly(I:C), TGF-β1, or mechanical compression

Poly(I:C) (Invivogen, San Diego, CA) or recombinant human (rh) TGF-β1 (10 ng/ml, Cell Signaling Technologies, Danvers, MA) was spiked into the basolateral media of HBE cells in ALI cultures [26, 27]. PBS for poly(I:C) or sodium citrate for rhTGF-β1 was used as vehicle control. As previously described [23, 24, 32], HBE cells were subjected to mechanical compression at a magnitude of 30 cm H_2_O pressure for three hours. Time-matched controls were subjected to 0 cm H_2_O pressure. In experiments where a pharmacological inhibitor of TGF-β receptor 1, SB431542 was used (10 μM, Cell Signaling Technology), this was spiked to the basolateral medium at a final concentration of 10 μM, one hour prior to exposure to either stimulation. As a vehicle control for SB431542, 0.1% DMSO was used.

### Real-time quantitative PCR analysis and enzyme-linked immunosorbent assay (ELISA)

As previously described [24, 33], we performed real-time RT-qPCR using the primers listed in Table 1, and then calculated fold-change for *EDN1* normalized to *GAPDH* using the 2^−ΔΔCT^ method; we quantified the amount of ET-1 protein in basolateral media from HBE cells or in BALF from mice using an ELISA kit (R&D Systems, Minneapolis, MN).

**Table 1:** Primer sequences used for RT-qPCR

Genes	Primer Sequences	References
*EDN1*	Fwd: 5’-AGAGTGTGTCTACTTCTGCCA-3’ Rev: 5’-CTTCCAAGTCCATACGGAACAA-3’	MGH primer bank
*GAPDH*	Fwd: 5’TGGGCTACACTGAGCACCAG-3’ Rev: 5’-GGGTGTCGCTGTTGAAGTCA-3’	[34]

### RV-induced exacerbation of allergic airways disease (AAD)

Measurements of lung function parameters, as well as bronchoalveolar lavage fluid supernatants, were collected from historical studies previously published [35–39], which are described below.

All animal models were reviewed and approved by the Animal Care and Ethics Committee at the University of Newcastle on protocols A-2016-605 and A-2020-014. BALB/c mice (at 6–8 wks old) obtained from Australian Bioresources (Mossvale, NSW Australia) were used in accordance with the Animal Research: Reporting of In Vivo Experiments (ARRIVE) guidelines.

To induce AAD, mice were sensitized and challenged with either ovalbumin (OVA) or house dust mite (HDM) [35–39]. Non-sensitized and non-challenged mice received sterile endotoxin-free saline. Mock infection utilized UV-inactivated RV-A1.

#### OVA

In the first model of AAD, mice were sensitized with hen egg OVA (50 μg/200 μl in 1% alhydrogel) on day − 14 and day − 7 intraperitoneally and then challenged with low LPS OVA (50 μg/30 μl PBS) for 3 consecutive days (days − 2, − 1, and 0) intranasally. On day 0, 6 h after the final OVA challenge, mice were infected with 2.5 × 10^6^ TCID_50_/ml of RV-A1, or PBS, intranasally.

#### HDM

In the second model of AAD, mice were sensitized with HDM (50 μg/50 μl in sterile saline) intranasally for three consecutive days. Twelve days after administration of the last sensitization dose, mice were challenged intranasally with HDM (5 μg/50 μl PBS) once daily for four consecutive days. At 24 h after final HDM challenge, mice were infected with RV-A1 (50 μl containing 1 × 10^7^ TCID_50_/ml), or UV-inactivated RV intranasally.

### Airway hyperresponsiveness

At 24 h after infection, mice were anesthetized for assessment of AHR and bronchoalveolar lavage fluid (BALF) samples were collected as previously described [37–39]. ET-1 protein in BALF was assessed by ELISA (R&D Systems) and correlated with previously published AHR data [37–39]. Airway resistance was expressed as a percentage change over baseline.

### Collection of bronchoalveolar lavage fluid

Upon euthanasia, mouse tracheas were cannulated, and lower airways flushed with HBSS (HycloneTM, GE Life Sciences). Cells were pelleted from lavage fluids and supernatants were used for measuring ET-1, as described above. To determine the correlation between ET-1 concentration in BALF and airway resistance, values of these two parameters were matched for each mouse.

### Statistical analysis

Statistical analysis was performed using GraphPad Prism 9 software (San Diego, CA). In experiments comparing two groups, a two-tailed Student’s t-test was used. In experiments comparing three or more groups, a one-way ANOVA or a mixed model was fitted with Tukey's correction for multiple comparisons (in vitro) or Holm-Sidak’s correction for multiple comparisons (in vivo). For calculating the correlation between ET-1 concentration and airway resistance, a Pearson correlation coefficient was calculated. A p-value less than 0.05 was considered statistically significant.

## Results

### In well-differentiated HBE cells, RV infection and poly(I:C) induce EDN1 expression and ET-1 secretion

**Fig. 1:**
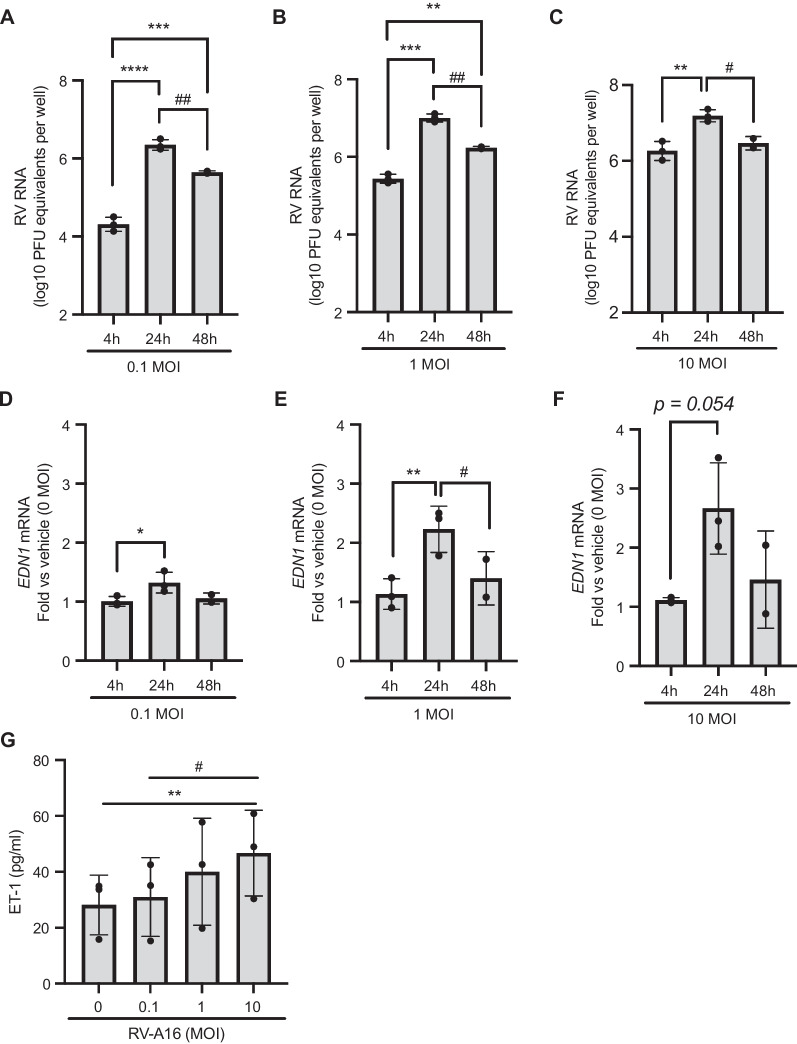
Rhinovirus infection induces ET-1 production. RV-A16 infection increased RV RNA in a dose-dependent manner (**A**–**C**). *p < 0.05, **p < 0.01, ***p < 0.001 vs 4 h, ^#^p < 0.05, ^##^p < 0.01 vs 24 h. RV-A16 infection induced *EDN1* expression (**D**–**F**, *p < 0.05 and **p < 0.01 vs 4 h, ^#^p < 0.05 vs 24 h) and ET-1 secretion (**G**, **p < 0.001 vs 0 MOI, ^#^p < 0.05 vs 0.1 MOI) in a dose-dependent manner. Each data point represents the mean of duplicate transwells of differentiated HBE cells from each donor (mean ± SD, n = 3 distinct donors)

Because ET-1 causes ASM cell contraction, as a potent bronchoconstrictor, we hypothesize that ET-1 could be a potential link between viral infection and bronchoconstriction. To determine the role of RV in ET-1 secretion, we infected HBE cells in air–liquid interface culture with increasing doses of RV-A16 (at MOI of 0.1, 1, or 10). Following a previously published protocol [40], we confirmed the productive infection of HBE cells by measuring replication of RV RNA [40]. RV RNA detected at 4 h post-infection (hpi) reflected the input of RV at each dose (Fig. 1A). Compared to the level of RV RNA detected at 4 hpi in each of the three doses, RV RNA was significantly increased (up to 2 logs) by 24 hpi (Fig. 1A–C). The increased RV RNA then slightly decreased by 48 hpi**.** At these three time points, we then measured mRNA expression of *EDN1* (the gene encoding ET-1 protein). At any of the three doses of RV, *EDN1* mRNA expression at 4 hpi was not different from *EDN1* mRNA expression of mock-infected cells as reflected by fold-changes of ~ 1 (Fig. 1D–F). At 24 hpi, infection with RV at MOI of 0.1 showed a modest but significant increase in *EDN1* expression (1.3-fold) compared to 4 hpi (*p < 0.05, Fig. 1D). Infection with the next dose of RV (at MOI of 1) significantly increased *EDN1* expression at 24 hpi by 2.0-fold compared to 4 hpi (**p < 0.01, Fig. 1E). The increased *EDN1* expression detected at 24 hpi was transient, returning to baseline by 48 hpi. With the highest infectious dose of RV (at MOI of 10) we tested, we also detected increased *EDN1* expression at 24 hpi by 2.4-fold compared to 4 hpi (p = 0.054, Fig. 1F). We then next measured secreted ET-1 protein in the basolateral media by ELISA at 24 hpi, to coincide with peak *EDN1* expression induced by RV infection. RV infection (at MOI of 10) significantly induced basolateral secretion of ET-1 compared to mock-infected cells (*p < 0.01, Fig. 1G) and cells infected with RV at MOI of 0.1 (^#^p < 0.05, Fig. 1G).

**Fig. 2:**
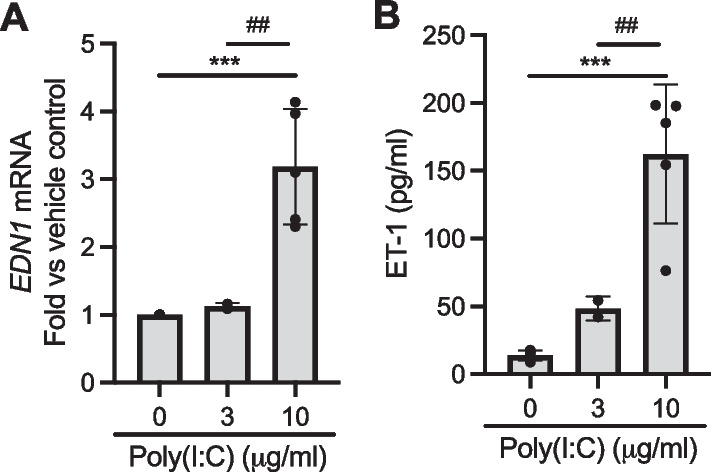
Poly(I:C), a TLR3 agonist, induces ET-1 production. Poly(I:C) induced *EDN1* expression **A** and ET-1 secretion **B** in a dose-dependent manner. ***p < 0.001 vs vehicle control, ^##^p < 0.01 vs 3 μg/ml. Each data point represents the mean of duplicate transwells of differentiated HBE cells from each donor (mean ± SD, n = 5 distinct donors)

Given our dose-dependent data demonstrating that RV at either MOI of 1 or 10 led to the significant replication of RV and induction of *EDN1* expression and ET-1 secretion (Fig. 1), we chose the dose of RV at MOI of 5, between 1 and 10, for the succeeding experiments. Having observed maximal RV-induced *EDN1* expression and ET-1 secretion at 24 hpi, we used this time point in additional studies to investigate mechanisms of ET-1 secretion from HBE cells. Within the infected cells, RV replication generates double-stranded RNA, which then activates toll-like receptor 3 (TLR3) [41, 42]. To determine if TLR3 activation alone is sufficient to induce ET-1 secretion by HBE cells, we incubated HBE cells with a TLR3 agonist, poly(I:C) (at 0, 3 or 10 μg/ml) for 24 h [41, 43]. Compared to vehicle control, poly(I:C) (at 10 μg/ml) significantly induced *EDN1* expression (***p < 0.001, Fig. 2A) and ET-1 secretion (***p < 0.001, Fig. 2B). Like RV infection, poly(I:C) treatment showed a dose-dependent induction of ET-1 secretion from HBE cells.

### RV-induced ET-1 secretion depends on the activation of the TGF-β receptor

**Fig. 3:**
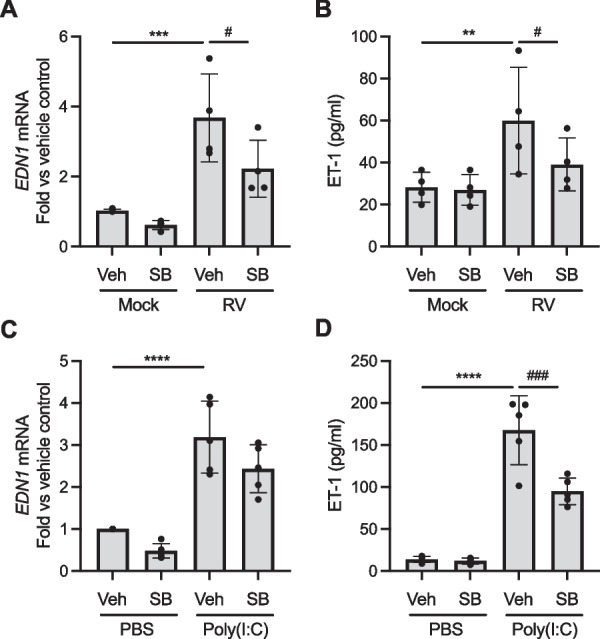
RV-induced ET-1 secretion depends on the activation of the TGF-β receptor. Pretreatment with SB431542 attenuated RV*-induced EDN1* expression (**A**) and ET-1 secretion (**B**). **p < 0.01 and ***p < 0.001 vs vehicle; ^#^p < 0.05 vs RV alone. Pretreatment with SB431542 decreased poly(I:C)-induced *EDN1* expression (**C**) and ET-1 secretion (**D**). ****p < 0.0001 vs vehicle; ^###^p < 0.001 vs poly(I:C) alone. Each data point represents the mean of duplicate transwells of differentiated HBE cells from each donor (mean ± SD; A, B: n = 4 distinct donors, C, D: n = 5 distinct donors)

To elucidate signaling pathways that regulate ET-1 synthesis and secretion, we investigated the potential role of the TGF-β receptor. TGF-β receptor activation increases viral replication [44] in HBE cells and TGF-β1 induces ET-1 secretion in rat type II alveolar epithelial cells [16], suggesting that the TGF-β receptor activation is a mechanism of RV-induced ET-1 secretion. To determine the necessity of TGF-β receptor activation for RV-induced ET-1 secretion, we blocked the activity of TGF-β receptor using a pharmacological inhibitor of TGF-β receptor, SB431542 (*denoted as SB*). RV infection (at MOI of 5) significantly induced *EDN1* expression and secretion in the absence of SB (Fig. 3A, B). Then, SB pretreatment significantly attenuated RV-induced *EDN1* expression (^#^p < 0.05, Fig. 3A) and RV-induced ET-1 secretion (^#^p < 0.05, Fig. 3B). In the same vein, pretreatment with SB decreased poly(I:C)-induced *EDN1* expression (Fig. 3C) and significantly attenuated poly(I:C)-induced ET-1 secretion (^###^p < 0.001, Fig. 3D).

### TGF-β receptor activation is both necessary and sufficient to induce epithelial ET-1 secretion

**Fig. 4:**
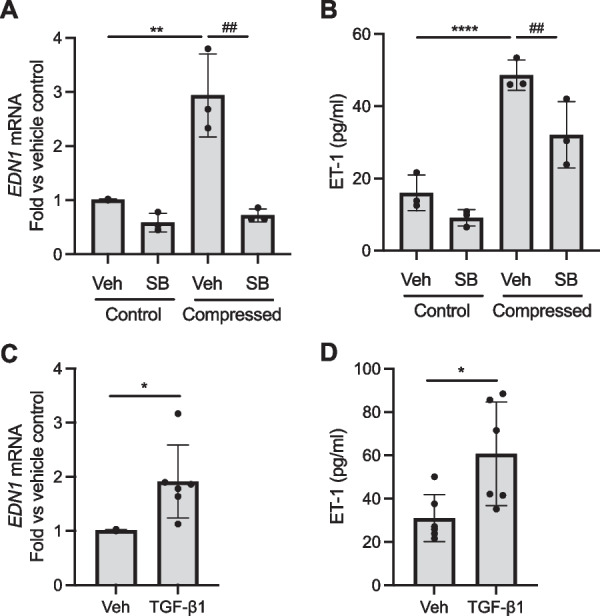
TGF-β receptor activation is necessary and sufficient to induce ET-1 secretion. Pretreatment with SB431542 attenuated compression-induced *EDN1* expression **A** and ET-1 secretion (**B**). **p < 0.01, ****p < 0.0001 vs vehicle; ^##^p < 0.01 vs compression alone. rhTGF-β1 (10 ng/ml) induced *EDN1* expression **C** and ET-1 secretion **D** *p < 0.05 vs vehicle. Each data point represents the mean of duplicate transwells of differentiated HBE cells from each donor (mean ± SD; **A**, **B**: n = 3 distinct donors, **C**, **D**: n = 6 distinct donors)

Given our new data demonstrating that RV induced ET-1 secretion through the activation of TGF-β receptor, we tested whether a non-viral stimulus, mechanical compression, also induced ET-1 secretion through TGF-β receptor. Consistent with our previous findings [23, 24], mechanical compression significantly induced *EDN1* expression (**p < 0.01, Fig. 4A) and ET-1 secretion (****p < 0.0001, Fig. 4B) above sham control. Pretreatment with SB significantly attenuated *EDN1* expression (^##^p < 0.01, Fig. 4A) and ET-1 secretion (^##^p < 0.01, Fig. 4B), both of which were induced by mechanical compression. To determine if the activation of TGF-β receptor alone was sufficient to induce ET-1 secretion from HBE cells, we treated HBE cells with rhTGF-β1 protein (at 10 ng/ml) for 24 h. Compared to vehicle control, rhTGF-β1 treatment significantly increased *EDN1* expression (*p < 0.05, Fig. 4C) and ET-1 secretion (*p < 0.05, Fig. 4D).

### In mouse models of AAD, RV-induced exacerbations are accompanied by increased ET-1 in BALF, correlating with AHR

**Fig. 5:**
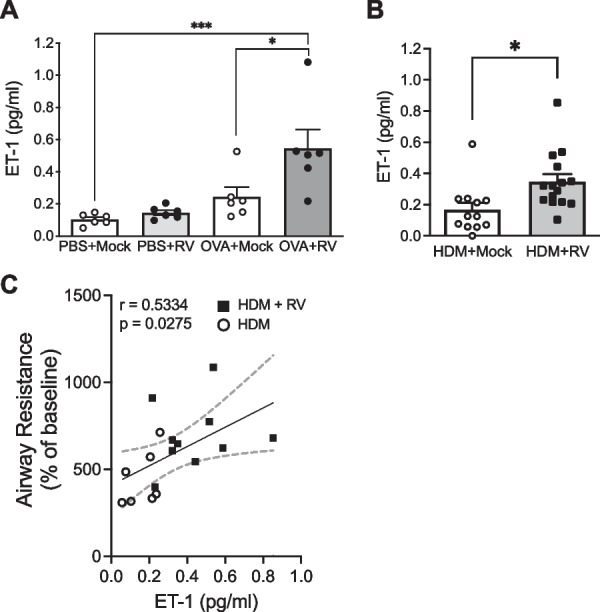
In mouse models of AAD, RV increased ET-1 concentration in BALF, correlating with AHR. In OVA-induced **A** or HDM-induced **B** model of AAD, RV infection increased ET-1 secretion in BALF collected at 24 hpi. *p < 0.05 vs OVA or HDM alone, ***p < 0.001 vs PBS + Mock; Mean ± SEM, n = 6 (**A**) and n = 12–15 (**B**). ET-1 concentration in BALF was correlated with AHR (**C**), n = 7–10 mice

To investigate a mechanistic link between ET-1 secretion and RV-induced bronchoconstriction in vivo, we utilized data and samples from two well-established mouse models of AAD sensitized and challenged with experimental allergens, either OVA or HDM [36, 38, 39, 45]. From these models, we measured ET-1 protein in BALF collected after mock or RV infections. In both OVA-allergic (*p < 0.05, Fig. 5A) and HDM-allergic (*p < 0.05, Fig. 5B) mice, RV infection significantly increased ET-1 measured in BALF over allergen challenge alone. To extend these findings, we tested for associations between ET-1 concentration in BALF and AHR and found a positive correlation (r = 0.5334, p = 0.0275, Fig. 5C).

## Discussion

Despite extensive epidemiological data associating RV infection with asthma exacerbations, the mechanisms of virus-induced bronchoconstriction and relationship with airway hyperresponsiveness are not fully understood. To investigate a potential mechanistic link of RV infection to asthma exacerbations, in particular, bronchoconstriction provoked through the action of the infected airway epithelial cells, we focused on the epithelial cell secretion of ET-1, a potent bronchoconstrictor. Since the discovery of ET-1 production by endothelial cells, mechanisms regulating its production have been extensively studied in endothelial cells because of its association with PAH [12]. However, regulatory mechanisms of ET-1 production may be disease-dependent and cell type-specific [13]. In the lung, ET-1 is prominently expressed in bronchial epithelial cells, which could be a source of secreted ET-1 in lung diseases. In our previous studies, we identified HBE cells as a source of secreted ET-1 [23, 24], which then significantly induces ASM contraction [24]. We, therefore, sought to identify mechanisms by which bronchial epithelial cells synthesize and secrete ET-1 in the context of RV-induced bronchoconstriction. Our data reveal that ET-1 secretion was induced by RV infection, TLR3 activation, or mechanical compression through the activation of the TGF-β receptor and that TGF-β receptor activation itself was sufficient to induce ET-1 secretion. We then extended our in vitro findings to an in vivo system using two mouse models of RV-induced asthma exacerbation, in which AAD is established by sensitization and challenge with OVA or HDM followed by sequential infection with RV. In both of these AAD models, RV significantly increased levels of ET-1 detected in BALF. The increased ET-1 in BALF correlated with AHR, suggesting a mechanistic link between ET-1 and airway responsiveness.

In well-differentiated HBE cells cultured from non-diseased donors, RV RNA was increased in a time- and dose-dependent manner (Fig. 1A–C). The kinetics of increased RV RNA were similar to the temporal changes of cell-associated viral RNA, which have been previously reported in HBE cells from non-diseased donors [40]. Similar to the kinetics of RV replication, RV-induced ET-1 production was increased in a time- and dose-dependent manner (Fig. 1D–G). Our data also demonstrate that poly(I:C), an agonist of TLR3 and viral mimic, induced ET-1 production in a dose-dependent manner (Fig. 2A, B). These results suggest that viral replication and TLR3 activation may be a mechanism for increased ET-1 secretion from HBE cells infected with RV. The TLR3-ET-1 axis could be a general mechanism of induction of bronchoconstriction when airway epithelial cells are infected with respiratory RNA viruses that can activate TLR3.

We further dissected intracellular signaling pathways by which ET-1 production is increased in airway epithelial cells. In endothelial cells or inflammatory cells, ET-1 expression is induced by various cytokines, such as IL-1β, TNF-α, and TGF-β [46]. Among them, we speculated a potential link between TGF-β1 and ET-1. In the lung, besides pulmonary hypertension, ET-1 is most well-studied in the context of pulmonary fibrosis, where TGF-β plays a prominent role in the pathogenesis of the disease [12, 13]. In patients with IPF, ET-1 is increased in serum and BALF and cellular expression of ET-1 is increased in airway epithelial cells and type II alveolar epithelial cells [15]. An in vitro study using rat type II alveolar epithelial cells demonstrated that TGF-β1 induces ET-1 secretion [16]. Moreover, TGF-β receptor activation leads to the reduction of antiviral responses while promoting viral replication in HBE cells, suggesting a potential role for the TGF-β receptor in the modulation of cellular responses during RV infection [44]. However, in this earlier study, RV infection did not directly activate SMAD determined by the detection p-SMAD, despite the inhibitory effect of TGF-β receptor on the viral replication suggesting that the baseline activity of TGF-β receptor is sufficient for airway epithelial cells to respond to RV infection. Thus, we examined the role of TGF-β receptor signaling for ET-1 secretion by blocking TGF-β receptor activity using a pharmacological inhibitor of TGF-β receptor or by activating TGF-β receptor activity using TGF-β1. Pre-treatment with SB, a TGF-β receptor inhibitor, significantly attenuated *EDN1* expression and ET-1 secretion, both of which were otherwise increased by RV infection or poly(I:C) treatment (Fig. 3). Like the effect of TGF-β receptor inhibition on ET-1 secretion that is induced by RV or poly(I:C), SB pretreatment also attenuated ET-1 secretion that is induced by mechanical compression (Fig. 4A, B). In our previous studies, we demonstrated the role of TGF-β pathways in mechanically compressed HBE cells. For example, RNA sequencing analysis revealed that TGF-β pathways are enriched in HBE cells by mechanical compression [47]; and TGF-β receptor signaling is partially required for compression-induced goblet cell hyperplasia [32]. Our new data here indicate that TGF-β receptor activation significantly contributed to compression-induced ET-1 secretion, which is a process relevant to bronchoconstriction as airway narrowing causes mechanical compression of HBE cells. Together, our in vitro data reveal that the activation of the TGF-β receptor on HBE cells is partially required for both viral and non-viral induction of ET-1 secretion from HBE cells. Thus, RV infection in combination with mechanical compression caused by bronchoconstriction may further augment secretion of ET-1 through the activation of TGF-β receptor, potentially leading to prolonged bronchoconstriction. Moreover, our data highlight the significance of crosstalk between RV infection and mechanical stimulation of airway epithelial cells in RV-induced asthma exacerbations. For example, a recent study demonstrated that mechanical compression suppresses antiviral innate immune responses from asthmatic airway epithelial cells following RV infection [48]. For a better understanding of virus-induced bronchoconstriction and its link with airway hyperresponsiveness, how these two factors, viral infection and mechanical compression, interact may be a major area of research for patients with RV-induced asthma exacerbations.

To determine the requirement of the TGF-β receptor in ET-1 secretion, we used SB431542 at 10 μM concentration that completely abolished the TGF-β receptor activity, as assessed by detection of p-SMAD2/3 in response to TGF-β1 (data not shown). However, complete inhibition of TGF-β receptor activity partially attenuated ET-1 synthesis or secretion that is induced by each of the three stimuli (Figs. 3, 4A, B). While blocking of TGF-β receptor activity led to the partial attenuation of increased ET-1 secretion, our data also indicate that TGF-β1 alone (Fig. 4C, D) was sufficient to induce ET-1 secretion. Our data further suggest that increased ET-1 secretion mediated by TGF-β receptor pathway could be a common mechanism for bronchoconstriction that is caused by infections with respiratory viruses (in addition to RV) that are known to induce TGF-β1, such as RSV [7, 49]. Moreover, comparative transcriptome analyses revealed that *EDN1* is one of the 43 hub genes induced by SARS-CoV-2 and also induced by other respiratory viruses, including human influenza viruses [50], suggesting that ET-1 may play additional roles in pathogenic processes such as inflammation and pulmonary fibrosis. In addition to virus-induced exacerbations, TGF-β1-induced ET-1 secretion from bronchial epithelial cells may constitute a novel pathway leading to bronchoconstriction during an asthma exacerbation either subsequent to or independent of viral infections. An OVA-induced AAD model in SMAD2 overexpressing mice suggests the potential role of TGF-β1 receptor in linking augmented ET-1 expression in airway epithelial cells and AHR [51]. In patients with asthma, TGF-β1 is increased in the lung and the most abundant source of TGF-β1 could be either injured epithelial cells, myofibroblasts, or active eosinophils [52]. Among these potential cellular sources of TGF-β1, considering the substantial role of eosinophils in asthma, more studies are necessary to determine whether TGF-β1 secreted by eosinophils directly stimulate airway epithelial cells to produce ET-1, which then exacerbates bronchoconstriction in asthma.

Given our in vitro data demonstrating that RV infection of airway epithelial cells induces the secretion of ET-1, a potent bronchoconstrictor, we aimed to determine if increased ET-1 is linked to RV-induced bronchoconstriction in vivo*.* We utilized samples and data from two well-established mouse models of AAD using sensitization and challenge with experimental allergens, OVA or HDM [36, 38, 39, 45]. In both OVA and HDM AAD models, where we have previously observed that infection with RV-A1 induces acute exacerbations [36, 38, 39, 45], RV infection significantly increased ET-1 measured in BALF over allergen challenge alone (Fig. 5A, B). Furthermore, when we pooled data on AHR from previous studies to match our ET-1 measurements from BALF [38, 39, 45], we found a significant correlation between AHR as measured by airway resistance and ET-1 concentrations in BALF (Fig. 5C). These in vivo data extend our in vitro findings by linking ET-1 secretion to RV-induced bronchoconstriction and AHR. Because AHR is a characteristic feature of asthma, our data support the hypothesis that airway epithelial cell-expressed ET-1 contributes to airway narrowing during RV-induced exacerbations of asthma. While ET-1 could be one of several mediators of bronchoconstriction, including histamine and leukotrienes [53, 54], here we demonstrate that ET-1 secreted from airway epithelial cells could be a critical mediator for virus-induced bronchoconstriction.

## Conclusions

In summary, our data reveal that RV infection of HBE cells induces secretion of ET-1 (a potent bronchoconstrictor) potentially through the sensing of double-stranded viral RNA by TLR3. Moreover, we demonstrate that both viral infection and mechanical compression induced ET-1 secretion from epithelial cells through the activation of the TGF-β receptor. Our data reveal that the activation of TGF-β receptor is not only required but also sufficient to induce ET-1 secretion in HBE cells. Given the effect of ET-1 on the contraction of ASM cells, this may be a novel mechanism mediating virus-induced bronchoconstriction. Mechanical compression of airway epithelial cells could further induce secretion of ET-1. In mouse models of AAD, increased ET-1 concentration in BALF correlated with AHR, both of which are characteristic features of asthma. Together the results from our in vitro and in vivo studies suggest that enhanced ET-1 secretion by airway epithelial cells may be a major driver of bronchoconstriction during RV-induced asthma exacerbations.

## Data Availability

All data generated or analyzed during this study are included in this manuscript.

## Abbreviations

AAD: Allergic airway disease
AHR: Airway hyperresponsiveness
ASM: Airway smooth muscle
BALF: Bronchoalveolar lavage fluid
ELISA: Enzyme-linked immunosorbent assay
ET-1: Endothelin-1
HBE cells: Human bronchial epithelial cells
HPI: Hours post-infection
HDM: House dust mite
MOI: Multiplicity of infection
OVA: Ovalbumin
Poly(I:C): Polyinosinic:polycytidylic acid
qRT-PCR: Quantitative reverse transcription polymerase chain reaction
RSV: Respiratory syncytial virus
RV: Rhinovirus
SARS-CoV: Severe acute respiratory syndrome coronavirus
SARS-CoV-2: Severe acute respiratory syndrome coronavirus 2
TGF-β: Transforming growth factor beta
TLR3: Toll-like receptor 3
